# Supplementary material for: Role and mechanism of NCAPD3 in promoting malignant behaviors in gastric cancer
Source: Front Pharmacol. 2024 Apr 22;15:1341039. doi: 10.3389/fphar.2024.1341039 (PMC11070777; doi:10.3389/fphar.2024.1341039)
Supplement: Supplementary file 11 [file DataSheet2.ZIP › GSEA/GO_CC/my_analysis.Gsea.1599461408869/GO_SUPRAMOLECULAR_COMPLEX.html]

Details for gene set GO\_SUPRAMOLECULAR\_COMPLEX[GSEA]

|  || Dataset | filtered\_dataset.sample\_info.cls#WT\_versus\_NCAPD3\_MUT |
| Phenotype | sample\_info.cls#WT\_versus\_NCAPD3\_MUT |
| Upregulated in class | NCAPD3\_MUT |
| GeneSet | GO\_SUPRAMOLECULAR\_COMPLEX |
| Enrichment Score (ES) | -0.22891104 |
| Normalized Enrichment Score (NES) | -1.7336739 |
| Nominal p-value | 0.0029498525 |
| FDR q-value | 0.11248648 |
| FWER p-Value | 0.486 |
Table: GSEA Results Summary

  

Fig 1: Enrichment plot: GO\_SUPRAMOLECULAR\_COMPLEX      
 Profile of the Running ES Score & Positions of GeneSet Members on the Rank Ordered List

  

| SYMBOL | TITLE | RANK IN GENE LIST | RANK METRIC SCORE | RUNNING ES | CORE ENRICHMENT || 1 | 342184 | FMN1 | 9 | 1.110 | 0.0128 | No |
| 2 | 7170 | TPM3 | 24 | 1.018 | 0.0202 | No |
| 3 | 10513 | APPBP2 | 28 | 1.000 | 0.0357 | No |
| 4 | 9759 | HDAC4 | 46 | 0.925 | 0.0391 | No |
| 5 | 23322 | RPGRIP1L | 60 | 0.871 | 0.0446 | No |
| 6 | 55794 | DDX28 | 68 | 0.856 | 0.0545 | No |
| 7 | 2926 | GRSF1 | 75 | 0.849 | 0.0650 | No |
| 8 | 56288 | PARD3 | 99 | 0.790 | 0.0615 | No |
| 9 | 51512 | GTSE1 | 127 | 0.774 | 0.0546 | No |
| 10 | 51174 | TUBD1 | 153 | 0.730 | 0.0485 | No |
| 11 | 7248 | TSC1 | 158 | 0.718 | 0.0582 | No |
| 12 | 6191 | RPS4X | 196 | 0.682 | 0.0421 | No |
| 13 | 9456 | HOMER1 | 198 | 0.680 | 0.0534 | No |
| 14 | 22948 | CCT5 | 215 | 0.663 | 0.0529 | No |
| 15 | 54801 | HAUS6 | 219 | 0.660 | 0.0624 | No |
| 16 | 9931 | HELZ | 229 | 0.651 | 0.0671 | No |
| 17 | 57521 | RPTOR | 239 | 0.645 | 0.0716 | No |
| 18 | 5701 | PSMC2 | 244 | 0.636 | 0.0799 | No |
| 19 | 324 | APC | 281 | 0.607 | 0.0632 | No |
| 20 | 4281 | MID1 | 294 | 0.595 | 0.0646 | No |
| 21 | 10426 | TUBGCP3 | 402 | 0.535 | -0.0074 | No |
| 22 | 259266 | ASPM | 411 | 0.531 | -0.0041 | No |
| 23 | 805 | CALM2 | 423 | 0.518 | -0.0033 | No |
| 24 | 801 | CALM1 | 424 | 0.518 | 0.0059 | No |
| 25 | 23242 | COBL | 426 | 0.516 | 0.0143 | No |
| 26 | 27097 | TAF5L | 461 | 0.498 | -0.0028 | No |
| 27 | 23116 | FAM179B | 482 | 0.486 | -0.0094 | No |
| 28 | 23468 | CBX5 | 484 | 0.485 | -0.0016 | No |
| 29 | 51112 | TRAPPC12 | 504 | 0.471 | -0.0077 | No |
| 30 | 1660 | DHX9 | 510 | 0.469 | -0.0032 | No |
| 31 | 27229 | TUBGCP4 | 556 | 0.442 | -0.0296 | No |
| 32 | 55308 | DDX19A | 615 | 0.416 | -0.0664 | No |
| 33 | 84948 | TIGD5 | 619 | 0.413 | -0.0614 | No |
| 34 | 26065 | LSM14A | 636 | 0.405 | -0.0664 | No |
| 35 | 6905 | TBCE | 652 | 0.399 | -0.0707 | No |
| 36 | 23093 | TTLL5 | 684 | 0.386 | -0.0875 | No |
| 37 | 5311 | PKD2 | 708 | 0.372 | -0.0984 | No |
| 38 | 203068 | TUBB | 736 | 0.358 | -0.1126 | No |
| 39 | 23741 | EID1 | 741 | 0.355 | -0.1094 | No |
| 40 | 10726 | NUDC | 754 | 0.346 | -0.1124 | No |
| 41 | 84148 | KAT8 | 757 | 0.344 | -0.1078 | No |
| 42 | 57619 | SHROOM3 | 783 | 0.331 | -0.1210 | No |
| 43 | 1605 | DAG1 | 823 | 0.304 | -0.1453 | No |
| 44 | 11157 | LSM6 | 830 | 0.296 | -0.1446 | No |
| 45 | 84823 | LMNB2 | 833 | 0.293 | -0.1409 | No |
| 46 | 23435 | TARDBP | 842 | 0.272 | -0.1422 | No |
| 47 | 4691 | NCL | 843 | 0.270 | -0.1374 | No |
| 48 | 200315 | APOBEC3A | 866 | -0.278 | -0.1492 | No |
| 49 | 9582 | APOBEC3B | 868 | -0.278 | -0.1451 | No |
| 50 | 6385 | SDC4 | 871 | -0.282 | -0.1416 | No |
| 51 | 667 | DST | 886 | -0.303 | -0.1469 | No |
| 52 | 27230 | SERP1 | 892 | -0.306 | -0.1453 | No |
| 53 | 9181 | ARHGEF2 | 902 | -0.326 | -0.1463 | No |
| 54 | 23710 | GABARAPL1 | 913 | -0.341 | -0.1479 | No |
| 55 | 301 | ANXA1 | 914 | -0.342 | -0.1419 | No |
| 56 | 84056 | KATNAL1 | 917 | -0.346 | -0.1372 | No |
| 57 | 4052 | LTBP1 | 928 | -0.353 | -0.1386 | No |
| 58 | 2317 | FLNB | 936 | -0.360 | -0.1376 | No |
| 59 | 8881 | CDC16 | 963 | -0.383 | -0.1506 | No |
| 60 | 80336 | PABPC1L | 971 | -0.387 | -0.1490 | No |
| 61 | 23499 | MACF1 | 989 | -0.398 | -0.1549 | No |
| 62 | 7277 | TUBA4A | 997 | -0.404 | -0.1531 | No |
| 63 | 83896 | KRTAP3-1 | 1006 | -0.408 | -0.1520 | No |
| 64 | 23046 | KIF21B | 1034 | -0.433 | -0.1649 | No |
| 65 | 81631 | MAP1LC3B | 1060 | -0.452 | -0.1759 | No |
| 66 | 7168 | TPM1 | 1086 | -0.465 | -0.1867 | No |
| 67 | 6795 | AURKC | 1100 | -0.477 | -0.1881 | No |
| 68 | 8444 | DYRK3 | 1131 | -0.502 | -0.2021 | No |
| 69 | 7074 | TIAM1 | 1143 | -0.508 | -0.2015 | No |
| 70 | 55124 | PIWIL2 | 1154 | -0.516 | -0.1999 | No |
| 71 | 1540 | CYLD | 1162 | -0.530 | -0.1959 | No |
| 72 | 1282 | COL4A1 | 1165 | -0.534 | -0.1879 | No |
| 73 | 3887 | KRT81 | 1168 | -0.536 | -0.1799 | No |
| 74 | 3866 | KRT15 | 1214 | -0.589 | -0.2038 | No |
| 75 | 114907 | FBXO32 | 1248 | -0.625 | -0.2178 | Yes |
| 76 | 63971 | KIF13A | 1256 | -0.645 | -0.2117 | Yes |
| 77 | 8651 | SOCS1 | 1272 | -0.661 | -0.2114 | Yes |
| 78 | 55638 | SYBU | 1275 | -0.663 | -0.2012 | Yes |
| 79 | 286 | ANK1 | 1284 | -0.672 | -0.1954 | Yes |
| 80 | 51175 | TUBE1 | 1286 | -0.677 | -0.1842 | Yes |
| 81 | 100820829 | MYZAP | 1291 | -0.682 | -0.1751 | Yes |
| 82 | 54464 | XRN1 | 1301 | -0.703 | -0.1695 | Yes |
| 83 | 7414 | VCL | 1308 | -0.712 | -0.1615 | Yes |
| 84 | 3892 | KRT86 | 1313 | -0.724 | -0.1517 | Yes |
| 85 | 57553 | MICAL3 | 1318 | -0.732 | -0.1417 | Yes |
| 86 | 6262 | RYR2 | 1323 | -0.741 | -0.1316 | Yes |
| 87 | 6509 | SLC1A4 | 1330 | -0.752 | -0.1229 | Yes |
| 88 | 10611 | PDLIM5 | 1335 | -0.758 | -0.1125 | Yes |
| 89 | 80709 | AKNA | 1351 | -0.795 | -0.1098 | Yes |
| 90 | 4622 | MYH4 | 1355 | -0.811 | -0.0977 | Yes |
| 91 | 3860 | KRT13 | 1367 | -0.847 | -0.0911 | Yes |
| 92 | 5630 | PRPH | 1371 | -0.885 | -0.0777 | Yes |
| 93 | 25819 | NOCT | 1374 | -0.891 | -0.0634 | Yes |
| 94 | 6840 | SVIL | 1377 | -0.916 | -0.0487 | Yes |
| 95 | 10397 | NDRG1 | 1380 | -0.925 | -0.0339 | Yes |
| 96 | 6711 | SPTBN1 | 1381 | -0.925 | -0.0175 | Yes |
| 97 | 8867 | SYNJ1 | 1387 | -0.979 | -0.0039 | Yes |
| 98 | 493 | ATP2B4 | 1401 | -1.166 | 0.0069 | Yes |
Table: GSEA details [plain text format]

  

Fig 2: GO\_SUPRAMOLECULAR\_COMPLEX      
 Blue-Pink O' Gram in the Space of the Analyzed GeneSet

  

Fig 3: GO\_SUPRAMOLECULAR\_COMPLEX: Random ES distribution      
 Gene set null distribution of ES for **GO\_SUPRAMOLECULAR\_COMPLEX**
